# Case report: term birth after fertility-sparing treatments for stage IB1 small cell neuroendocrine carcinoma of the cervix

**DOI:** 10.1186/s12905-017-0404-0

**Published:** 2017-07-28

**Authors:** Pei-Ying Wu, Ya-Min Cheng, Geok Huey New, Cheng-Yang Chou, Chun-Ting Chiang, Hung-Wen Tsai, Yu-Fang Huang

**Affiliations:** 1 Department of Obstetrics & Gynecology, National Cheng Kung University Hospital, College of Medicine, National Cheng Kung University, 138, Sheng-Li Rd. Northern District, Tainan, 70403 Taiwan; 2 Department of Pathology, National Cheng Kung University Hospital, College of Medicine, National Cheng Kung University, 138, Sheng-Li Rd. Northern District, Tainan, 70403 Taiwan

**Keywords:** Fertility-sparing, Small cell neuroendocrine carcinoma, Cervical carcinoma, Radical trachelectomy, Chemotherapy, Pregnancy and delivery, Case report

## Abstract

**Background:**

Advances in cervical cancer management for childbearing women have led to less radical approaches. Use of fertility-sparing treatment to treat small cell neuroendocrine carcinoma (SCNEC) is challenging owing to the aggressive nature of the disease, even in early stage disease.

**Case presentation:**

A 25-year-old nulligravida woman presented with malodorous vaginal discharge and was diagnosed to have an exophytic cervical SCNEC. A magnetic resonance image scan showed no evidence of parametrial invasion or distant metastasis. Clinical staging allocated her to stage IB1 disease. She underwent radical abdominal trachelectomy for reproductive purpose. Preoperative and postoperative chemotherapy with ifosfamide/etoposide/cisplatin combining gonadotropin-releasing hormone agonist were administered. She had a spontaneous, uneventful pregnancy and successfully delivered a term baby via cesarean section 7 years after treatment.

**Conclusion:**

To our knowledge, we describe the first success in offering a fertility-preserving multimodality strategy to present favorable oncologic, reproductive, and obstetric outcomes in a fertile woman of stage I SCNEC. Individualized multimodality therapy may be utilized in specific patients with early-stage cervical cancer to preserve their fertility.

## Background

Small cell neuroendocrine carcinoma (SCNEC) comprises 1–3% of cervical carcinoma and is the most common of four neuroendocrine tumors, as delineated according to the College of American Pathologists and the National Cancer Institute [1]. SCNEC is characterized pathologically by a high mitotic rate, extensive necrosis, and frequent lymphovascular space involvement. SCNEC spreads aggressively, with frequent hematogenous metastasis, locoregional recurrence, and short survival, even in patients with early-stage disease [2]. Use of chemotherapy regimens, similar to those used to treat small cell lung cancer (SCLC), after radical surgery or accompanying radiation improves survival of patients with SCNEC [3, 4].

SCNEC treatment is more challenging than that for other histological subtypes because most patients have poor prognoses, even those with early-stage disease. The 10-year overall survival rate is 55% for patients with SCNEC, compared to 76% for those with adenocarcinoma and 88% for those with squamous cell carcinoma (SCC) among the International Federation of Obstetrics and Gynecology (FIGO) stage IB1 cervical carcinoma [2]. The 5-year overall survival (OS) rates for patients with early-stage SCNEC or SCC were 30.5% and 98.0%, respectively, whereas the 5-year progression-free survival rates were 26.5% and 94.0%, respectively [5]. Patients with early-stage SCNEC may be more susceptible to treatment failure because of the high rates of lymphovascular space invasion that result in hematogenous spread or lymph node metastasis [5, 6].

Nowadays, fertility-sparing treatment in patients with early-stage cervical carcinoma who are of reproductive age is an important issue. Promising reproductive outcomes after uterine conservation treatments have been reported [7, 8]. However, the available data for these procedures did not include SCNEC cases for analysis. Therefore, the National Comprehensive Cancer Network guidelines have not yet made a recommendation regarding fertility-sparing modalities to treat SCNEC [9]. A recent case report described a multiparous woman with microscopically diagnosed stage IB1 SCNEC after cold knife conization [10]. She underwent an abdominal radical trachelectomy (ART) and bilateral pelvic lymphadenectomy, followed by adjuvant chemotherapy. No cancer recurrence or pregnancy occurred during the following 26-month surveillance. However, a role for neoadjuvant chemotherapy (NACT) in treatment of patients with SCNEC who have tumors larger than 2 cm has not yet been described in the literature. Here, we report the case of a patient with SCNEC who had a successful term birth 7 years after peri-operative fertility-sparing treatment for FIGO stage IB1 disease (>2 cm) without evidence of cancer recurrence.

## Case presentation

**Fig. 1:**
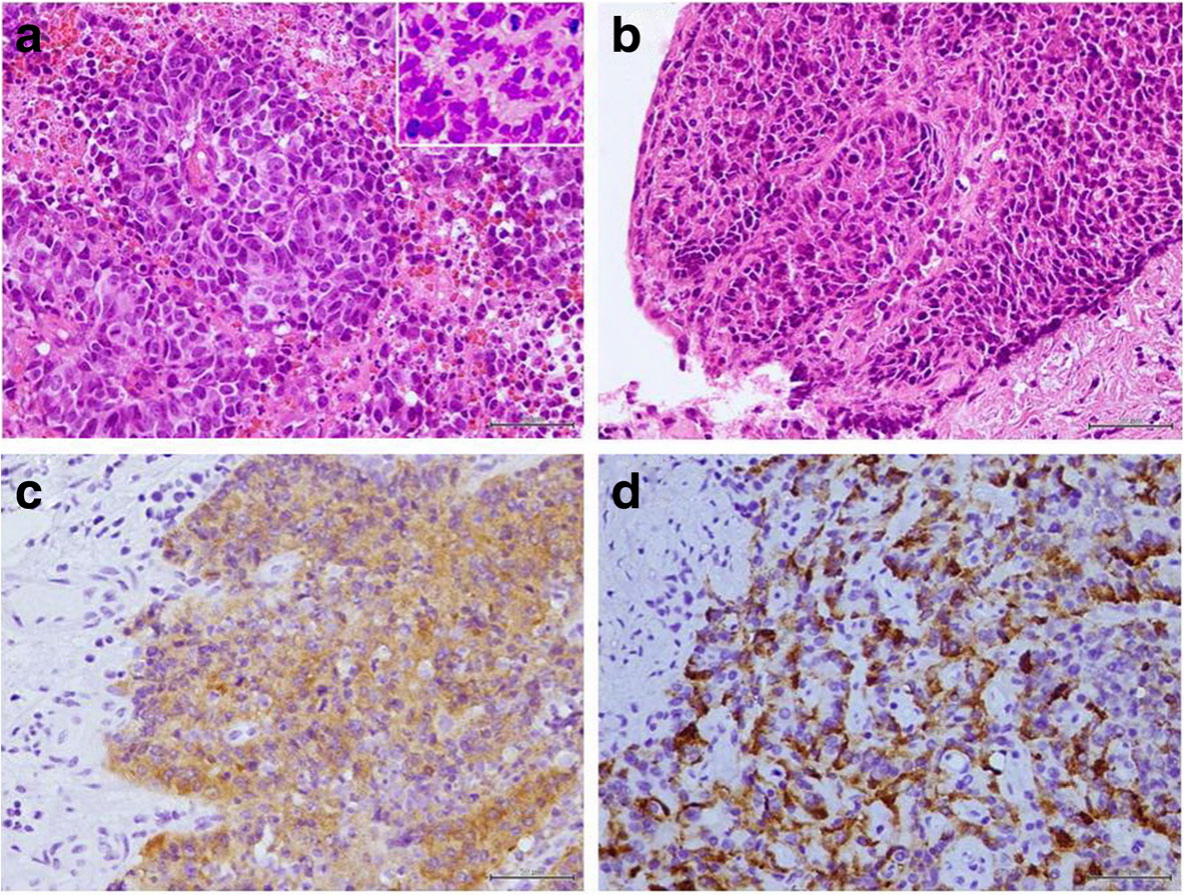
Microscopic examination of hematoxylin and eosin-stained tissue sections (**a**) before and (**b**) after neoadjuvant chemotherapy. The tumor appears as an exophytic mass with stromal invasion and comprises small cells with scant cytoplasms, round-to-oval nuclei with a salt-and-pepper appearance, and abundant mitotic figures. Tumor cells stained positively for **(c)** synaptophysin and **(d)** chromogranin A

A 25-year-old nulligravida woman presented with 5-month intermittent vaginal spotting and a large amount of malodorous vaginal discharge. She had no history of smoking, medical diseases, or family history of malignancy. No previous cervical smear and human papillomavirus (HPV) DNA test have been obtained. On pelvic examination, a 3.0-cm exophytic, fragile cervical tumor, was identified. No parametrial or vaginal involvement was detected. A colposcopy-directed cervical biopsy was performed, and tissue was submitted to pathology. Microscopic examination showed a poorly differentiated carcinoma composed of small cells with scant cytoplasms, oval nuclei with a salt-and-pepper appearance, and abundant mitotic figures (>20 mitoses per high power field) (Fig. 1). Immuno- histochemical stains revealed that this tumor was positive for synaptophysin and chromogranin A, which are markers of a neuroendocrine tumor.

**Fig. 2:**
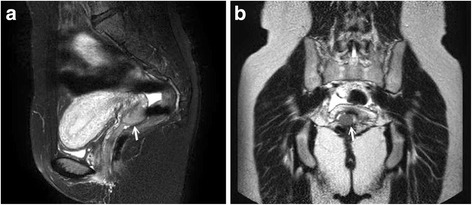
Pelvic magnetic resonance imaging findings prior to neoadjuvant chemotherapy. The lesion measured approximately 2.7 × 1.8 × 1.5 cm and was located over the right side of the cervix. The tumor was confined to the cervix, with no invasion to the adjacent tissue, parametria, or vagina. **a** A sagittal plane, T2-weighted image shows hyperintensity with fat saturation and gadolinium enhancement. The repetition time (TR) was 2966, and the echo time (TE) was 70. **b** A coronal view T2-weighted image shows an intermediate signal lesion, with a TR of 692 and a TE of 150

Chest radiography, abdominal sonography, and whole body bone scintigraphy were all negative for cancer metastasis. She had elevated CA-125 level (47.1 IU/mL) and normal levels of CA-199 and carcinoembryonic antigen. She did not have paraneoplastic syndrome (hyponatremia, hypoosmolarity and hypercalcemia). Pretreatment neurological exam revealed normal neurological function. Central nerve system (CNS) metastasis was not suspected, because no clinical evidence of CNS involvement was encountered. A pelvic magnetic resonance image (MRI) scan (Fig. 2) showed an exophytic cervical lesion (2.7 cm in diameter) that had not invaded the parametria, adjacent organs, or regional lymph nodes. Clinical staging resulted in a diagnosis of stage IB1 SCNEC.

She was appropriately counseled to ensure that she had realistic expectations with respect to various issues regarding preoperative chemotherapy, postsurgical fertility, cytotoxic chemotherapy or radiation, and cancer prognosis. We perceived her strong desire to become pregnant. She was administered two cycles of NACT with ifosfamide/etoposide/cisplatin (IEP), and the tumor had shrunk to less than 2 cm prior to radical surgery. We then performed surgery, including ART, dissection of bilateral pelvic lymph nodes, and endometrial curettage. The specimen was proximally excised 5 mm below the internal os and sent for frozen section examination to ensure safe surgical margins at the endocervix and parametria. The vaginal mucosa was reapproximated to the neocervix with interrupted sutures, and nonresorbable monofilament 0-polypropylene sutures were used to perform cerclage. A cervical stent was transcervically inserted during the operation to avoid cervical stenosis and removed 3 weeks later. Pathologic examination revealed that the cervical tumor was exophytic with superficial stromal invasion (4 mm in depth) and no lymphovascular space invasion. The endocervix, parametria, vaginal end, and 19 lymph nodes were all negative for malignancy.

The patient was administered 6 cycles of IEP chemotherapy postoperatively, along with gonadotropin-releasing hormone agonist (GnRHa) to minimize the risk of oocyte loss. Post-treatment physical examinations were performed every 3 months for the first 2 years, every 4 months for the third year, every 6 months for the fourth and fifth years, and annually thereafter, based on the recommendations of the Society of Gynecologic Oncologists [11]. Neurological exam, cervical cytology and serum marker evaluations at the same time. All clinical assessments for cancer recurrence yielded negative results during the post-treatment surveillance period.

Pregancy was allowed 6 months after completion of adjuvant chemotherapy. The patient had regular menstrual cycles and underwent no artificial reproductive technique prior to her first pregnancy, which was reported 6.5 years post-treatment. The pregnancy proceeded uneventfully. Surveillance examinations were performed every 3 months during pregnancy. We performed a cesarean section at 39 weeks of gestation to deliver a female baby weighing approximately 3.2 kg. The patient recovered rapidly and was discharged 5 days after cesarean delivery without events. Breastfeeding efforts began within the first hour after birth in the operating room. She resumed monthly menstrual cycles 2 months later.

## Conclusion

We demonstrate the first successful fertility-sparing treatment in a woman with a FIGO stage IB1 SCNEC tumor larger than 2 cm that resulted in long-term survival as well as favorable oncologic and reproductive outcomes. In general, SCNEC is considered an extra-pulmonary variant of SCLC [12]. These cancer cells may extensively spread to the liver, adrenals, bone, lymph nodes, and brain to cause associated symptoms and body weight loss. Paraneoplastic syndrome, including syndromes of inappropriate antidiuretic hormone secretion, Cushing syndrome, and hypercalcemia, may also be observed concomitantly [13]. There is a strong correlation between SCNEC and HPV-18 infection [14]. In most cases, the disease is highly aggressive, with an early propensity for hematogenous spread, and frequently fatal, even in patients with early-stage disease.

Unfavorable prognostic factors of SCNEC include advanced stage at diagnosis [3, 4], no chemotherapy or chemoradiation use [3], and lymph node metastasis [4]. Adjuvant chemotherapy has been proposed for SCNEC in any stage. Poor clinical outcomes in patients with advanced-stage SCNEC and SCC after treatment are similar [5]. Primary concurrent chemoradiation with ≥5 cycles of etoposide/cisplatin (EP)-containing chemotherapy was found to provide survival benefits in patients with stage IIB–IV disease [4]. The most common approach to treat early-stage SCNEC is radical hysterectomy with regional lymphadenectomy followed by chemotherapy or chemoradiation [4, 12]. However, the low 5-year survival due to treatment failure, ranging from 36.8% to 51.5%, in patients with stage I–IIA SCNEC is disappointing [3, 4]. Lee et al. reported that adjuvant chemoradiation was not superior to adjuvant chemotherapy alone in early-stage SCNEC patient survival [15]. Moreover, pelvic radiation is not preferred for women who want to preserve ovarian function. This suggests that innovative multimodality treatment, including combined systemic therapy, should be investigated to improve treatment efficacy.

Use of fertility-sparing surgery to treat cervical cancers has become widely accepted owing to a growing body of evidence on surgical safety as well as satisfactory oncologic and pregnancy outcomes [7, 8]. The information on outcomes is limited to cases with common cervical carcinoma histology. Radical trachelectomy (RT), a valid alternative to radical hysterectomy, can be performed vaginally, abdominally, or endoscopically in patients with FIGO stage IA−IB cervical cancer (lesion ≤2 cm without extension to internal os/low segment) who want to preserve fertility [16–18]. The abdominal route is optimal when vaginal access is not possible in nulligravida women, as it allows surgeons to obtain more parametrium than that via the vaginal route. The overall conversion rate from ART to radical hysterectomy was 10% of 485 patients with common pathology in a systematic review [17]. Approximately 9% of patients had cervical stenosis [8, 17], and the recurrence rate after ART was 0–4.8%, while the pregnancy rate after ART was 15.5–44% [7, 8]. The safety and feasibility of laparoscopic and robotic RT have been documented [18]. Compared with ART, laparoscopic and robotic approaches result in less blood loss, shorter hospital stays, comparable cancer recurrence rates, and similar operative outcomes [19, 20]. Pregnancy rates are higher in patients who underwent ART than in those who underwent minimally invasive surgery. However, data for long-term oncologic outcome are still unavailable in common and uncommon histology.

Physicians should be alert when patients with early-stage cervical cancer demonstrate high risks for recurrence or mortality. Pretreatment surveys should include assessments of tumor size, lower uterine segment/endocervical involvement, and parametrial and nodal infiltration. MRI is a useful and recommended tool to ascertain the aforementioned parameters in addition to detecting disease high in the endocervix, which cannot be identified via physical examination. Therefore, MRI helps to guide decisions regarding fertility-sparing versus non-fertility-sparing treatment approaches [21]. Other imaging modalities, such as positron emission tomography/computed tomography scanning and next-generation MRI techniques for preoperative assessment of microscopic lymph node involvement are still under investigation.

NACT is thought to provide benefits to enhance the resectability of bulky tumors with negative margins, and it may prevent hematogenous spread before surgery. NACT may allow surgeons to preserve the uterus in young patients who initially do not fulfill the strict tumor size criteria for primary radical surgery. Both oncological and pregnancy outcomes after NACT and fertility-preserving surgical procedures, including ART, vaginal radical trachelectomy, simple trachelectomy, and large cone biopsy, have been reviewed [22]. No convincing data have helped identify ideal surgical procedures after NACT because of insufficient patient numbers and variety in the cytotoxic agents used. Recurrence rates after NACT range 7–20%. The impact that NACT has on survival is inconclusive owing to a lack of long-term results. The incidence of cervical stenosis in patients receiving NACT is 6.2–25%, which requires the use of assisted reproduction. The pregnancy rate is 14.3−50% in patients who were administered NACT, with intrauterine infection and premature rupture of the membrane being the most common antepartum risks; moreover, preterm delivery due to these complications occurs in 28.6−50% of pregnancies. Spontaneous pregnancies are rare in patients treated with NACT plus ART, and most patients require reproductive procedures. All studies investigating RT alone or NACT plus RT excluded unusual histological subtypes, such as sarcoma, neuroendocrine, papillary serous, and clear cell carcinoma. Currently, there is no available information on the use of NACT followed by less radical surgery in SCNEC.

Adjuvant chemotherapy regimens for SCNEC treatment are different from those for SCC treatment. The EP doublet is the most commonly used chemotherapeutic regimen [3, 6]. To systemically control SCNEC, regimens vary by adding doxorubicin or ifosfamide to the EP regimen or by adding irinotecan or paclitaxel/ifosfamide to a cisplatin-based regimen, in addition to vincristine/adriamycin/cyclophosphamide [4, 23]. In a multi-institutional retrospective study, clinicopathological and treatment variables related to prognosis in 179 patients with SCNCC were investigated [4]. Stage I patients had a significantly lower risk for cancer death than stage II patients, reemphasizing the importance of early cancer detection. The 5-year cancer-specific and failure-free survival rates in patients who received peri-operative chemotherapy plus surgery were 53.3% and 60.0%, respectively, whereas the survival rates in those who received NACT plus surgery were merely 16.7% and 16.7%, respectively. This may suggest that the combination of chemotherapy and NACT with surgery improves survival rates of patient with early-stage SCNEC.

The use of GnRHa during chemotherapy in cancer survivors has been shown to advantageously preserve ovarian function [24], with significantly protective effects that have been demonstrated in multiple randomized trials of breast cancer [25]. All studies indicate GnRHa coadministration during chemotherapy remarkably reduces the premature ovarian failure rate from 33.5% to 18.5%. In our case, cyclic ovarian function was preserved after GnRHa coadministration with 6 cycles of three cytotoxic agents.

With updated imaging modalities and advances in peri-operative chemotherapeutic regimens as well as uterine preservation surgical techniques, gynecologic oncologists could provide multimodality therapy to young women with tumors of rare histology who wish to preserve their fertility. In conclusion, fertility-sparing surgery may be individualized in specific cases after detailed evaluation and appropriate counseling. This approach deserves future investigation to examine its feasibility in patients with stage I SCNEC.

## Abbreviations

ART: Abdominal radical trachelectomy
EP: Etoposide/cisplatin
FIGO: International Federation of Obstetrics and Gynecology
GnRHa: Gonadotropin-releasing hormone agonist
IEP: Ifosfamide/etoposide/cisplatin
MRI: Magnetic resonance image
NACT: Neoadjuvant chemotherapy
OS: Overall survival
RT: Radical trachelectomy
SCC: Squamous cell carcinoma
SCLC: Small cell lung cancer
SCNEC: Small cell neuroendocrine carcinoma
